# A Randomized Controlled Trial of a Brief Intervention for Delayed Psychological Effects in Snakebite Victims

**DOI:** 10.1371/journal.pntd.0003989

**Published:** 2015-08-11

**Authors:** Chamara A. Wijesinghe, Shehan S. Williams, Anuradhani Kasturiratne, Nishantha Dolawaththa, Piyal Wimalaratne, Buddhika Wijewickrema, Shaluka F. Jayamanne, Geoffrey K. Isbister, Andrew H. Dawson, David G. Lalloo, H. Janaka de Silva

**Affiliations:** 1 Faculty of Medicine, University of Kelaniya, Ragama, Sri Lanka; 2 District General Hospital, Polonnaruwa, Sri Lanka; 3 South Asian Clinical Toxicology Research Collaboration, Peradeniya, Sri Lanka; 4 Clinical Toxicology Research Group, University of Newcastle, Newcastle, NSW, Australia; 5 Prince of Wales Clinical School, University of New South Wales, Sydney, NSW, Australia; 6 Liverpool School of Tropical Medicine, Liverpool, United Kingdom; Emory University, UNITED STATES

## Abstract

**Background:**

Snakebite results in delayed psychological morbidity and negative psycho-social impact. However, psychological support is rarely provided to victims.

**Aim:**

To assess the effectiveness of a brief intervention which can be provided by non-specialist doctors aimed at reducing psychological morbidity following snakebite envenoming.

**Method:**

In a single blind, randomized controlled trial, snakebite victims with systemic envenoming [n = 225, 168 males, mean age 42.1 (SD 12.4) years] were randomized into three arms. One arm received no intervention (n = 68, Group A), the second received psychological first aid and psychoeducation (dispelling prevalent cultural beliefs related to snakebite which promote development of a sick role) at discharge from hospital (n = 65, Group B), while the third received psychological first aid and psychoeducation at discharge and a second intervention one month later based on cognitive behavioural principles (n = 69, Group C). All patients were assessed six months after hospital discharge for the presence of psychological symptoms and level of functioning using standardized tools.

**Results:**

At six months, there was a decreasing trend in the proportion of patients who were positive for psychiatric symptoms of depression and anxiety from Group A through Group B to Group C (Chi square test for trend = 7.901, p = 0.005). This was mainly due to a decreasing trend for symptoms of anxiety (chi-square for trend = 11.256, p = 0.001). There was also decreasing trend in the overall prevalence of disability from Group A through Group B to Group C (chi square for trend = 7.551, p = 0.006), predominantly in relation to disability in family life (p = 0.006) and social life (p = 0.005). However, there was no difference in the proportion of patients diagnosed with depression between the three groups (chi square for trend = 0.391, p = 0.532), and the intervention also had no effect on post-traumatic stress disorder.

**Conclusions:**

A brief psychological intervention, which included psychological first aid and psychoeducation plus cognitive behavioural therapy that can be provided by non-specialist doctors appeared to reduce psychiatric symptoms and disability after snakebite envenoming, but not depression or post-traumatic stress disorder.

**Trial Registration:**

Sri Lanka Clinical Trials Registry: SLCTR/2011/003

## Author Summary

Snakebite is an important health problem in many rural communities in tropical countries. However, little is known about the lasting physical and mental health effects following a bite. We recently reported that mental problems, with harmful social outcomes, can occur in many people after they are bitten by a snake. As the affected are often poor farmers or manual workers, this may affect their livelihoods. We, therefore, performed a trial which looked at the effectiveness of short psychological interventions, lasting about 15 and 20 minutes, which can be provided by even non-specialist doctors, in reducing these mental and social problems in people bitten by snakes. Our results show that such interventions may indeed be helpful to reduce some of these problems, but more research is needed to improve these interventions, especially so that they that can reduce post-traumatic stress disorder and depression after snakebite.

## Introduction

Snakebite causes significant morbidity and mortality and the highest burden exists in the poorer rural populations of South Asia, Southeast Asia, and sub-Saharan Africa. Globally, it has been estimated that snakebite results in as many as 1.8 million envenomings and 94 000 deaths each year [1]. In Sri Lanka, about 35,000 to 40,000 persons are treated in hospital for snakebite each year [2]. The actual number of bites is likely to exceed this number, as some victims seek traditional forms of treatment and do not come into contact with mainstream health services. The affected are often poor farmers or manual workers in whom the snakebite may result in loss of livelihood and good health [3].

Though the physical impacts of snakebite are well documented and researched, the long term psychological impact of snakebite remained largely unexplored. We recently reported that significant delayed psychological morbidity occurs in victims of snakebite, including increased rates of depression, anxiety and post-traumatic stress disorder (PTSD), with associated negative psychosocial impact [4]. This study showed that snakebite victims had more depressive symptoms than controls based on the modified Beck Depression Scale and more symptoms of depression and anxiety measured by the Hopkins Symptoms Checklist. Fifty four per cent of the cohort met criteria for depressive disorder compared to only 15% of the controls. PTSD occurred in 22% of patients and 27% claimed that the snakebite caused a negative change in their employment with 10% stopping work and 17% reporting residual physical disability. This is hardly surprising, as the affected are often poor farmers or manual workers in whom the snakebite may result in loss of livelihood [4].

There are many myths surrounding snakebite. In many Asian and African cultures snakes are considered deities and hence snakebite can be misconstrued as punishment from the gods. These myths are not merely confined to Asia and Africa as is evident by the snake receiving prominence in most western health emblems due to its perceived mythical prowess and impact on health [5]. To this day in Sri Lanka, a ritualistic dance called the “*sanninatuma*” is performed to exorcise the demons causing eighteen common illnesses, and one of these dances specifically targets “*Naga Sanniya*” which literally translates to “snake madness” and is characterized by nightmares involving snakes and inability to perform activities of daily living [6]. “*Naga sanniya*” may be the first recorded description of psychological disability following snakebite. The physical impact of snakebite can be coloured by cultural beliefs, and it is not uncommon for people to believe that snakebite will lead to sapping of strength, diminished physical abilities, blindness, physical disfiguration and an overall inability to function at their premorbid level leading to avoiding work, withdrawing from social life and resigning themselves to a life of suffering [4].

Improved emergency care, availability of antivenom, and increasing numbers of victims seeking hospital based care has led to a reduction in mortality rates and physical complications of snake bite [7]. However, until recently, delayed psychological morbidity following snakebite was not recognized and psychological support is rarely offered to victims, although its inclusion into snakebite management protocols has been recommended [7,8].

Psychoeducation, which refers to the education offered to individuals with a mental health condition and their families to help empower them and deal with their condition in an optimal way, has been shown to be of benefit in the treatment of many mental conditions [9]. Although there are no precedents for the use of psychoeducation following animal bites, there is evidence for the usefulness of psychoeducation following traumatic situations [10]. Trauma based cognitive behavioural therapy is also recommended in the treatment of mental illness following stressful life events [11]. There is evidence to suggest that brief interventions based on trauma focused cognitive behavioural therapy are effective in the prevention of PTSD [12].

The aim of this study was to assess the effectiveness of a brief psychological intervention provided by non-specialist doctors in reducing delayed psychological morbidity and negative social impact associated with snakebite envenoming.

## Materials and Methods

This was a single blind, randomized, controlled, parallel design trial of a brief psychological intervention in snakebite victims to determine its effectiveness in reducing psychiatric symptoms, depression, psychosocial disability and PTSD.

### Ethics Statement

Ethical approval for the study was obtained from the Ethics Review Committee of the Faculty of Medicine, University of Kelaniya, Ragama. The study was registered as a clinical trial with the Sri Lanka Clinical Trials Registry (SLCTR/2011/003). All subjects provided written informed consent.

### Study Patients

The study was conducted in the Polonnaruwa District General Hospital in Sri Lanka from August 2011 to April 2014. Polonnaruwa is situated in the northeast of the country and has a predominantly rural agricultural population. The area has one of the highest rates of snakebite envenomings in the country [13]. All snakebite victims admitted to hospital identified as being envenomed and requiring treatment with antivenom were eligible for inclusion. Exclusion criteria were those under 18 years of age, those with known mental illness, and those without basic fluency in the Sinhala language.

### Study Protocol

After snakebite patients had received standard medical treatment for their snake envenoming, they were randomized to one of three study arms before discharge from hospital, after obtaining written informed consent. Group A received no psychological intervention, Group B received a psychological intervention based on psychological first aid and psychoeducation at time of discharge from hospital, and Group C received psychological first aid and psychoeducation at time of discharge and were subsequently recalled one month following discharge from hospital and provided with a psychological intervention based on trauma based cognitive behavioural therapy principles. All participants were assessed six months following discharge from hospital.

### Sample Size Calculation

A sample size of 195 (65 in each arm of the study) was calculated in order to detect a 50% reduction in rates of depressive disorder in the intervention group assuming an incidence of 54% as detected in our previous study [4], a power of 80% and a significance level of 0.05. A 10% loss to follow up rate was assumed, resulting in an increase in the sample size to 216. We selected depression for sample size calculation as it was an important disability that was identified in our previous study [4].

### Psychological Interventions

The brief interventions were administered by non-specialist doctors. The non-specialist doctors involved in the study were trained by a specialist psychiatrist, initially over a period of one week with continued support over the course of the study. They were trained on communication skills and counseling via practical demonstration.

#### Intervention at discharge focused on psychological first aid and psychoeducation

The intervention consisted of the doctor engaging in a focused discussion about the patient’s opinion on the causes and consequences of the snake bite. The intervention followed the normal doctor patient interview style in which patients were initially engaged in open ended questions and allowed to express their views. The conversation was then moved into a more structured discussion, using a structured check list to ensure a degree of standardization. This list included important thoughts to elicit, such as myths, negative assumptions, and future plans and expectations of the patient. If any erroneous or maladaptive ideas were identified, they were challenged in a non-confrontational manner and more plausible, evidence based alternative views were expressed. Common misbeliefs which have a negative impact on a person’s psychological adaptation and subsequent functional level were specifically addressed. Patients were encouraged to engage in a healthy lifestyle following discharge and to avoid the trap of assuming a sick role [14]. This intervention was provided in a secluded portion of the ward when the patient had recovered from the acute complications of snakebite and was ready for discharge. Adequate time was provided to express their feelings and concerns, and the intervention typically lasted 15 minutes. It was provided at an intermediate stage when the patient was physically stable and ready for discharge and was based more about facilitating adjustment to the event rather than on the snakebite itself.

#### Cognitive behavioural therapy (CBT) intervention

A trauma based cognitive behavioural therapy approach was used because snakebite is a severe, sudden and traumatic event.

In this single intervention one month post discharge, victims were initially engaged in a focused discussion on how they had functioned in their daily lives after the snakebite and whether they had any ongoing difficulties. A checklist guided the doctors providing the intervention to identify the victims’ dysfunctional cognitions related to health, personal life, functional abilities, and overall future expectations. If dysfunctional cognitions were elicited, they were reframed in a positive manner. The doctors also encouraged return to work and normal life. In patients who had not gone back to work to an optimal level, activity scheduling was introduced. A phased return to household and occupational activity was suggested and patients were encouraged to return to their hobbies and pleasurable activities. Other maladaptive coping methods such as substance misuse were discussed and counseling provided as needed. In situations where there was a understandable anxiety about returning to work and being bitten again by a snake, practical safety measures such as wearing boots and gloves and carrying a torch and a stick were encouraged. The duration of this intervention was typically 20 minutes.

### Data Collection—Post-Intervention Assessment

Patients were assessed for presence of psychological morbidity and functional status six months following discharge from hospital by a specialist psychiatrist blind to intervention status and trained in the usage of the study tools. Psychological distress was quantified using a number of measures: the Hopkins symptoms checklist– 25(HSCL-25) [15,16], a modified Sinhala version of the Beck depression inventory [17], the Sheehan Disability Inventory [18], and the Post-traumatic Stress Symptom Scale—Self Report (PSS-SR) [19]. All have been previously validated and used in Sri Lanka [20].

The Hopkins Psychiatric Symptom Checklist measures a combination of depressive and anxiety symptoms. It does not provide a diagnosis of illness but based on the score, classifies subjects as positive or negative for psychiatric symptoms. The Beck's modified depression scale scores were categorized into no depression (0–15), mild depression (16–24), moderate depression (25–32) and severe depression (>32) in terms of accepted figures. The established clinically significant item-average cut-off score of ≥1.75 for each sub-scale was used for the Hopkins somatic symptoms checklist. An overall cut-off of 15/30 and a domain specific cut-off of 5/10 were used for the Sheehan Disability Inventory. The generally accepted cut off score ≥20 on the PSS-SR was taken as compatible with post-traumatic stress disorder.

### Data Analysis

The outcomes assessed were the proportions of patients with psychiatric symptoms overall, positive symptoms of depression, positive symptoms of anxiety (based on the Hopkins Somatic Symptoms Checklist), depression (based on Beck’s Modified Depression Scale), disability in family life, social life and work (based on Sheehan’s Disability Inventory) and PTSD.

### Statistical Analysis

Analysis of quantitative data was done using SPSS version 16 on an intention to treat basis. Chi square test for trend and Fisher’s exact test were used to assess the differences between the study groups. After adjustment for multiple testing, a p<0.0125 was considered significant.

## Results

**Fig 1:**
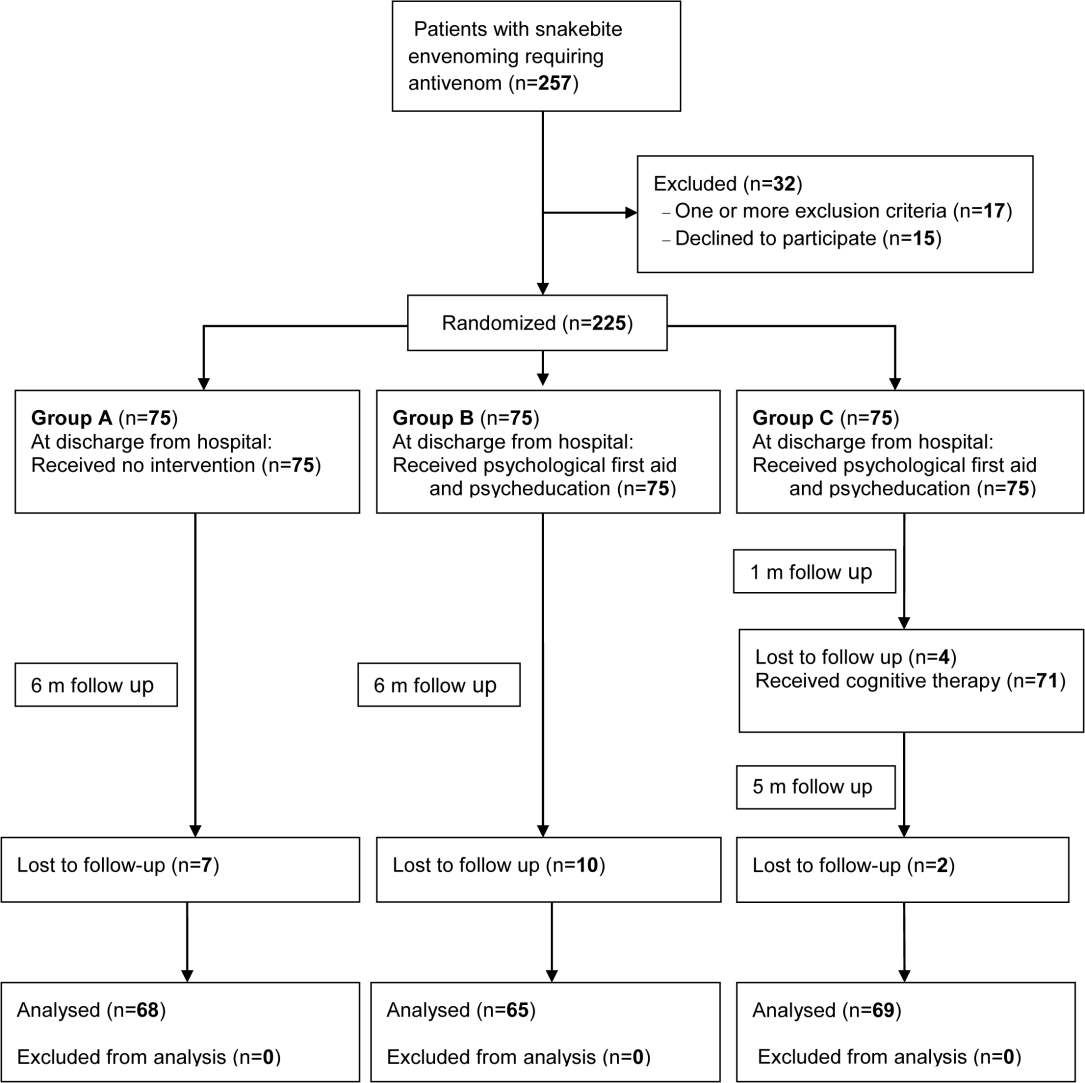
Study flow chart.

There were 225 snakebite victims [168 males, mean age 42.1 (SD 12.4) years] who were randomized into one of the three study arms (n = 75 each). Of these, 202 (89%) (Group A, n = 68; Group B, n = 65; Group C, n = 69) completed the study and were assessed at 6 months after discharge from hospital (Fig 1).

Male farmers of working age were highly represented in the study sample. There were no differences in age, sex or occupation between the three groups (Table 1).

**Table 1:** Comparison of demographic characteristics of the study groups (each group n = 75).

Variable	Group A	Group B	Group C	Significance
Age in years, Mean (SD)	42.5 (14)	41.3 (12.5)	40.2 (13.2)	NS#
Male (%)	55 (73.3)	57 (76.0)	56 (74.7)	NS**
Farming occupation (%)	41 (59.4)	42 (64.6)	39 (57.3)	NS**
Biting species identified	14	15	25	NS*
- *Daboia russelii*	*10*	*12*	*15*	
- *Naja naja*	*01*	*02*	*05*	
- *Bungarus caeruleus*	*03*	*01*	*05*	
Severe reactions to AVS	07	06	08	NS*
Treated in Intensive Care	02	02	01	NS*
- Mechanical ventilation	01	00	01	NS*
Acute renal failure (dialysed)	01	00	00	NS*
Tissue necrosis	01	00	03	NS*

# Student's t test

** Chi square test

* Fisher's exact test

Overall, the biting species was identified in 24.1%; the proportion of species identified was similar in the three groups. The proportion of patients who developed severe reactions to AVS [21] and who were treated in intensive care were similar in the three groups. Four patients developed tissue necrosis, but none required amputation.

At six months, the overall proportion of patients who were positive for psychiatric symptoms of depression and anxiety was 18/68 (26.5%) in Group A, compared to 9/65 (13.8%) in Group B and 6/69 (8.7%) in Group C. This decreasing trend was statistically significant (Chi square test for trend = 7.901, p = 0.005). On further analysis, this decreasing trend was seen for both symptoms of anxiety (Chi square test for trend = 11.256, p = 0.001), and symptoms of depression (Chi square test for trend = 5.793, p = 0.016) (Table 2).

**Table 2:** Results of the Hopkin’s psychiatric symptoms checklist among study groups.

Group	Symptoms		Significance
	Present (%)	Absent (%)	
**Total**			
**A (n = 68)**	**18 (26.5)**	**50 (73.5)**	**Test for trend**
**B (n = 65)**	**09 (13.8)**	**56 (86.2)**	**Chi Square = 7.901**
**C (n = 69)**	**06 (8.7)**	**63 (91.7)**	**p = 0.005**
**Total**	**33 (16.3)**	**169 (83.7)**	
Depression			
A (n = 68)	16 (23.5)	52 (76.5)	Test for trend
B (n = 65)	09 (13.8)	56 (86.2)	Chi Square = 5.793
C (n = 69)	06 (8.7)	63 (91.7)	p = 0.016
Total	31 (15.3)	171 (84.7)	
Anxiety			
A (n = 68)	18 (26.5)	50 (73.5)	Test for trend
B (n = 65)	09 (13.8)	56 (86.2)	Chi Square = 11.256
C (n = 69)	04 (5.8)	65 (94.2)	p = 0.001
Total	31 (15.3)	171 (84.7)	

Depression was diagnosed in 21/68 (30.9%) patients in Group A, 17/65 (26.2%) patients in Group B and 18/69 (26.1%) patients in Group C. These rates did not show a statistically significant trend (chi square for trend = 0.391, p = 0.532) (Table 3).

**Table 3:** Prevalence of depression among the study groups (BDI).

Group	Depression		
	Present (%)	Absent (%)	Significance
A (n = 68)	21 (30.9)	47 (69.1)	Test for trend
B (n = 65)	17 (26.2)	48 (73.8)	Chi Square = 0.391
C (n = 69)	18 (26.1)	51 (73.9)	p = 0.532
Total	56 (27.7)	146 (72.3)	

However, on further analysis, the rate of severe depression was significantly higher in Group A (10.3%) compared to Group B (1.5%) and Group C (0) (Fisher’s exact test p = 0.004) (Table 4).

**Table 4:** Severity of depression among the study groups (BDI).

Group	Severe Depression (%)	Moderate Depression (%)	Mild Depression (%)	No Depression (%)	Significance
A (n = 68)	7 (10.3)*	8 (11.8)	6 (8.8)	47 (69.1)	Fisher’s exact
B (n = 65)	1 (1.5)	7 (10.8)	9 (13.8)	48 (73.8)	test,
C (n = 69)	0 (0.0)	2 (2.9)	16 (23.2)	51 (73.9)	p = 0.004*
Total	8 (4.0)	17 (8.4)	31 (15.3)	146 (72.3)	

*Significant difference identified through item-wise analysis

The overall prevalence of disability was 18/68 (26.5%) in Group A compared to 11/65 (16.9%) in Group B and 6/69 (8.7%) in Group C with a statistically significant decreasing trend from Group A through Group B to Group C (chi square for trend = 7.551, p = 0.006; Table 5). On further analysis, this decreasing trend was seen in relation to disability in family life (p = 0.006) and social life (p = 0.005), but not in relation to disability at work (p = 0.056).

**Table 5:** Prevalence of disability among the study groups (SDI).

Type	Group	Disability		Significance
		Present (%)	Absent (%)	
**Overall disability**	**A**	**18 (26.5)**	**50 (73.5)**	**Chi square for**
	**B**	**11 (16.9)**	**54 (83.1)**	**trend = 7.551**
	**C**	**06 (8.7)**	**63 (91.3)**	**P = 0.006**
	**Total**	**35 (17.3)**	**167 (82.7)**	
Disability in family life	A	18 (26.9)	49 (73.1)	Chi square for trend = 7.490
	B	13 (20.0)	52 (80.0)	P = 0.006
	C	06 (8.7)	63 (91.3)	
	Total	37 (18.4)	164 (81.6)	
Disability in workplace	A	20 (29.4)	48 (70.6)	Chi square for trend = 3.640
	B	13 (20.0)	52 (80.0)	P = 0.056
	C	11 (15.9)	58 (84.1)	
	Total	44 (21.8)	158 (78.2)	
Disability in social life	A	17 (25.0)	51 (75.0)	Chi square for trend = 7.901
	B	11 (16.9)	54 (83.1)	P = 0.005
	C	5 (7.2)	64 (92.8)	
	Total	33 (16.3)	169 (83.7)	

The overall prevalence of PTSD was (17/202) 8.4%. The proportion of patients with PTSD was 7/68 (10.3%) in group A, compared to 8/65 (12.3%) in group B and 2/69 (2.9%) in Group C which was not statistically significant (Chi-square for trend = 2.448; p = 0.118) (Table 6).

**Table 6:** Prevalence of post-traumatic stress disorder among the study groups.

Group	PTSD		Significance
	Present (%)	Absent (%)	
A (n = 68)	07 (10.3)	61(89.7)	Test for trend
B (n = 65)	08 (12.3)	57 (87.7)	Chi Square = 2.448
C (n = 69)	02 (2.9)	67 (97.1)	p = 0.118
Total	17 (8.4)	185 (91.6)	

A sensitivity analysis was conducted assuming the worst possible scenario (worst clinical outcome) in the patients who dropped out after randomization without receiving the intervention and outcome assessment. The decreasing trend observed from Group A through Group B to Group C in the results of Hopkins Psychiatric Symptoms Checklist and Sheehan Disability Inventory remained statistically significant.

## Discussion

We found that brief psychological interventions by non-specialist doctors, which included psychological first aid and psycho-education at discharge and a single cognitive behavioural therapy based intervention one month after discharge, appeared to reduce psychiatric symptoms of anxiety and depression, and improve overall functionality, especially those related to family and social life in victims of snake bite envenoming. The interventions did not reduce the proportion of patients with PTSD or overall depression, but appeared to reduce severe depression. The apparent discrepancy in the fact that overall depression was not reduced though there was a reduction in severe depression in the intervention groups, may reflect the fact that the interventions are simply effective in converting severe depression to milder forms. Our findings have important implications given the potential the socio-economic burden that may result from psychological disability following snakebite envenoming in this predominantly subsistence farming population. The proportion of patients who developed severe reactions to AVS, who developed tissue necrosis and who were treated in intensive care were similar in the three groups. These factors are, therefore, unlikely to have influenced the differences in outcome between the three groups.

Psychological interventions following trauma aimed at preventing psychiatric illness have generally shown mixed results [14]. Trauma based cognitive behavioural therapy is recommended in the treatment of psychological disorders following stressful life events [11] because there is evidence to suggest that brief interventions based on trauma focused cognitive behavioural therapy is effective in the prevention of PTSD [12]. Recent studies also support single interventions such as ours as being useful [22].

A study on psychological interventions following trauma from Chile has shown reduction in rates of depression and improvement of functional levels, although PTSD rates did not improve [23]. Cognitive errors are known to dominate the thoughts of a person afflicted with secondary depression [24]. Therefore, they may be more easily targeted by providing psychological first aid and psychoeducation and cognitive behavioural therapy. However PTSD constitutes a constellation of symptoms less under cognitive control. This may not respond to brief psychological interventions. Rates of PTSD were not reduced by the interventions conducted in our study and this reflects the findings of studies conducted elsewhere [12,25], and underscores the view that one intervention may work better for a particular symptom but not for another. The challenge would be to design a brief intervention that is able to reduce all types of symptoms to some extent, although it would possibly not be the ideal intervention for each individual symptom.

Although there was a trend towards reduction of psychiatric symptoms as well as improved functionality in both intervention groups, the best outcome was seen in the group which received both psychological first aid and psychoeducation and the cognitive behavioral therapy based intervention. Though the interventions were designed on existing models and delivered in a structured manner, the factors that played a role in reducing psychological morbidity remain to be determined. Common therapeutic factors such as confidence in the therapist, explaining illness factors and interaction with the therapist have a positive effect regardless of type of psychotherapy provided [25]. We are unable to ascertain if it was the specific therapy provided or the interaction and counseling provided by a health care professional that caused the beneficial effect seen in our study. Further analysis of the interventions will be required to improve them further. Nevertheless, the fact that psychological interventions provided by non-specialist doctors seemed to help in reducing some psychological morbidity in snakebite victims is encouraging and warrants further exploration.

Psychological therapies are provided by health care professionals other than psychiatrists and psychologists. These include general practitioners, counsellors, therapists, social workers and psychiatric nurses. The lack of qualified psychotherapists is a worldwide problem [26]. Snakebites occur predominantly in poorer social settings in Asia and Africa, and so it is unlikely that specialist mental health services will be optimal in these settings. Training non-specialist doctors to provide brief psychological interventions may be a viable alternative option. Having a non-specialist doctor providing the psychological intervention as opposed to a psychiatrist or psychologist may even improve compliance, given the stigma associated with mental illness which remains a significant barrier towards accessing mental health care [27].

Our study has limitations. As ours was an intention to treat analysis, the dropout rate after randomization may have affected the results. However, there was no change in the overall results after a sensitivity analysis was conducted assuming the worst possible clinical outcome in the patients who dropped out after randomization without receiving the intervention and outcome assessment. Also, as more than one outcome of the intervention was assessed, multiple comparisons had to be made. However adjustments were made for multiple comparisons when calculating significance. Psychiatric caseness was not confirmed by a psychiatrist or by using detailed protocols, as we used only screening instruments for depression and anxiety. However, all of the instruments we used have been previously validated and used in Sri Lanka [20]. We did not have any information on pre-event rates of depression or anxiety in the study group which may have affected our results. In an attempt to minimize such an effect we excluded patients with known mental illness from the study. It is commonly believed that some snakes induce more fear and could be psychologically more traumatic than bites of other snakes. Although of potential interest, we were unable to perform a subgroup analysis of our results based on biting species as the offending snake was identified in a minority of cases. This is the usual situation in rural Sri Lanka where the offending snake is brought to hospital very infrequently, venom antigen detection is not routinely available, and bites often occur in situations where the snake is not even seen clearly—eg. in scrub jungle, paddy fields.

In conclusion, this study is the first attempt to treat the recently recognized problem of psychological morbidity following snakebite envenoming. The results clearly suggest a role for brief psychological interventions post-snake bite, provided by non-specialist doctors that should be achievable even in settings in which mental health services are sub-optimal. However, further research is required to refine the types of interventions, focusing on PTSD and depression.

## Supporting Information

S1 Checklist CONSORT checklist. (DOC)
